# Second Primary Malignancies in Patients With Hepatocellular Carcinoma: A Population-Based Analysis

**DOI:** 10.3389/fonc.2021.713637

**Published:** 2021-08-23

**Authors:** Junjie Kong, Guangsheng Yu, Wei Si, Guangbing Li, Jiawei Chai, Yong Liu, Jun Liu

**Affiliations:** ^1^Department of Liver Transplantation and Hepatobiliary Surgery, Shandong Provincial Hospital, Cheeloo College of Medicine, Shandong University, Jinan, China; ^2^Department of Liver Transplantation and Hepatobiliary Surgery, Shandong Provincial Hospital Affiliated to Shandong First Medical University, Jinan, China; ^3^Department of Breast and Thyroid Surgery, Shandong Maternity and Child Care Hospital, Jinan, China

**Keywords:** hepatocellular carcinoma, secondary primary malignancy, competing-risk model, nomogram, SEER

## Abstract

**Background:**

Second primary malignancy (SPM) is becoming a threat for the health of cancer survivors. However, data on the features and results of patients with hepatocellular carcinoma (HCC) with SPMs are scarce. This study aimed to explore the characteristics of HCC patients with SPMs and to screen HCC patients who are at a high risk of developing SPMs.

**Method:**

HCC patients diagnosed between 2000 and 2014 in the Surveillance, Epidemiology, and End Results (SEER) database were retrospectively analyzed. Eligible patients were divided into the only one primary malignancy and SPM groups. The Fine-Gray proportional subdistribution hazards model was used to explore the risk factors of developing SPMs, and a competing-risk model was established to predict the probability of developing SPMs for HCC patients after initial diagnosis. The calibration curves, concordance index (C-index), and decision curve analysis (DCA) were used to evaluate the performance of the nomogram.

**Results:**

A total of 40,314 HCC patients were identified, 1,593 (3.95%) of whom developed SPMs 2 months after the initial diagnosis with a maximum follow-up time of approximately 18 years. The 3-, 5-, and 10-year cumulative incidence of SPMs were 2.35%, 3.12%, and 4.51%, respectively. Age at initial diagnosis, extent of disease, tumor size, and treatment were identified as the independent risk factors of developing SPMs and integrated into the competing-risk nomogram. The C-index of the nomogram was 0.677 (95% confidence interval 0.676–0.678), and the calibration curves showed an excellent agreement between the nomogram prediction and the actual observations. Furthermore, DCA indicated that the nomogram had good net benefits in clinical scenarios.

**Conclusions:**

HCC survivors remain at a high risk of developing SPMs. The development of SPMs was associated with the clinical features and treatment strategies. A competing-risk nomogram was constructed to help surgeons identify the patients who are at a high risk of developing SPMs and contribute to the further management of SPMs.

## Introduction

Liver cancer (LC) is the sixth most commonly diagnosed malignancy and the third leading cause of cancer-related death worldwide (1). In 2020, it was reported that the number of new cases and deaths of LC in the world were 905,677 and 830,180, respectively (1). Hepatocellular carcinoma (HCC) is the most common type of LC, which accounts for 75%–85% of cases. With the advancements in early diagnosis, cancer treatment, and cancer surveillance, the prognosis of HCC has greatly improved (2). For HCC patients diagnosed at an early stage, some of them could even have a 5-year overall survival (OS) of nearly 70% after operation (3). However, among HCC patients with a long-term survival, the second primary malignancy (SPM) is becoming a threat for their lives, and the studies on this problem are urgent. Recently, a growing number of studies have been performed to explore the risk factors of developing SPMs in various cancers, such as colorectal cancer (4), lung cancer (5), gastric cancer (6), and breast cancer (7).

The occurrence of SPMs in HCC was first reported by Riesz et al. (8) in 1979. Afterwards, several studies were published to describe this problem (9–11); however, these studies were limited by the small number of patients and different study design. Recently, using the public database, two population-based studies were published to describe the development of SPMs in HCC (12, 13); however, the conclusions we could get from the two studies were limited. The study by Shan (13) only focused on the simple description of SPMs in HCC, while in the study by Wu (12), only four variables including age, sex, treatment, and comorbidities were included in the exploration of the risk factors of developing SPMs. Less is known about the influence of clinical features including race, laboratory data, and tumor characteristics (such as size, vascular invasion, and stage) on the development of SPMs in HCC.

In this study, using the data obtained from the Surveillance, Epidemiology, and End Results (SEER) research database, we aimed to comprehensively analyze the clinicopathological characteristics of HCC patients with SPMs and to explore the risk factors associated with the development of SPMs. Afterwards, a competing-risk nomogram was established to predict the probability of developing SPMs for HCC patients, and the Harrell’s concordance index (C-index), calibration curves, and decision curve analysis (DCA) were used to evaluate the performance of the nomogram.

## Materials and Methods

### Data Sources and Patient Selection

The SEER Research Data, 18 Registries (excl AK), Nov 2019 Sub (2000–2017) for Standardized Mortality Ratios (SMRs, https://seer.cancer.gov/) was used as the data source, and the “MP-SIR” session in the SEER*Stata software (Version 8.3.8) was used to extract related data. The SEER 18 Registry research data (excl AK) for SMRs consisted of patients from the San Francisco-Oakland, Connecticut, Detroit, Hawaii, Iowa, New Mexico, Seattle, Utah, Atlanta, San Jose-Monterey, Los Angeles, Rural Georgia, California, Kentucky, Louisiana, New Jersey, and Greater Georgia tumor registries, which covered approximately 27.8% of the US population. We screened patients diagnosed with HCC using the third edition of the International Classification of Diseases for Oncology (ICD-O-3) site codes C22.0 and the Hist/behave codes 8170/3, 8171/3, 8172/3, 8173/3, 8174/3, or 8175/3. The patients with death certificate of autopsy records were excluded, furthermore, a 2-month latency exclusion period was set to distinguish SPMs from simultaneous HCC. Patients who were initially diagnosed with HCC aged between 20 and 80 years old were included in this study. To ensure a follow up period for at least 3 years, patients initially diagnosed with HCC after 2014 were excluded. The work flow of patient selection is shown in **Figure S1**. Subsequently, patients were divided into two groups: SPM group and only one primary malignancy (OOPM) group.

### Definition of SPM

In the SEER database, the cancer site of origin, date of diagnosis, histology, tumor behavior, and laterality of paired organs were used to discriminate multiple primary cancers. Generally, we could define SPM as a neoplasm that arose independently in a new site or tissue and subsequent to the initial cancer, with the intervening period being at least 2 months (14). Furthermore, in the selection of patients, two key variables in the SEER database, “total number of *in situ*/malignant tumors for patient” and “sequence number” could indicate the occurrence of SPMs. The former could be used to screen patients with SPMs, and for HCC patients with SPMs, the sequence number for the first cancer was “01”, “02” for the second, and so on.

### Outcome and Covariables

The occurrence of SPMs after the initial diagnosis of HCC was studied. OS referred to the time from the initial diagnosis of HCC to any cause of death. SPM OS referred to the time from the diagnosis of SPMs to death of any cause. Patient-, tumor-, and treatment-related covariates included in the analysis of this study contained age at initial diagnosis (<50, 50–65, and 65–79), sex, race (white, black, and other), tumor grade (I/II, III/IV, and unknown), tumor extension (localized, regional, distant, and unknown), treatment (none, local treatment, hepatectomy, transplantation, and unknown), tumor size (0–2 cm, 2–5 cm, >5 cm, and unknown), vascular invasion (none, yes, and unknown), AFP level (negative, positive, and unknown), status (dead/alive), and cause of death (first primary tumor, multi malignancies, noncancer cause, and unknown).

### Statistical Analysis

Categorical variables were displayed as numbers (n) and percentages (%) and continuous variables as median and interquartile ranges (Q1–Q3). To compare the difference between different groups, the chi-square test or Fisher’s test was used for categorical variables and the Mann–Whitney U-test for continuous variables. Since the occurrence of death was a competitive event for the development of SPMs, the use of the Cox regression analysis could lead to an overestimation of the cumulative incidence of SPMs (15). Consequently, using the “cmprsk” R package, the Fine and Gray proportional subdistribution hazards model was used to estimate the cumulative incidence function (CIF) of the covariables on death and SPMs (16–18). Univariate analysis was conducted using the CIF to display the probability of each event and Gray’s test to evaluate the difference in the CIF between groups (19). Multivariate analysis with the Fine-Gray model was performed to identify the risk factors associated with the development of SPMs. The hazard ratio (HR) and the associated 95% confidence interval (CI) were recorded.

Based on the results of the Fine-Gray model, a competing-risk nomogram was established using the “rms” R package to predict the 3-, 5-, and 10-year probability of developing SPMs for HCC patients after initial diagnosis. The performance of the nomogram was evaluated using the Harrell’s concordance index (C-index), and the calibration curves were performed to assess the predictive accuracy of the nomogram using 200 bootstrap samples (20). Furthermore, using the “stdca” R package, DCA was conducted to evaluate the clinical benefit of the nomogram (21).

R software (version 4.0.3) was used in the statistical analysis of this study. All tests were two sided, and a p < 0.05 was considered statistically significant.

## Results

### Population Characteristics

**Figure 1:**
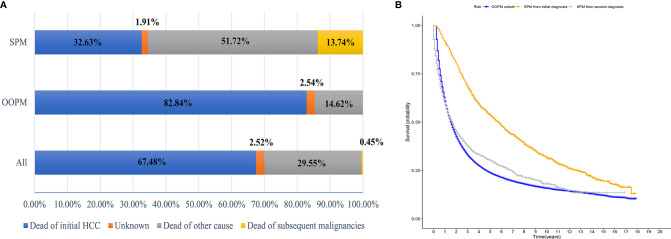
Distribution of the causes of death and estimates of overall survival for HCC patients. **(A)** Distribution of the causes of death for all HCC patients and for patients in the OOPM and SPM groups. **(B)** Estimates of overall survival for HCC patients in the OOPM group, for patients in the SPM group from their initial diagnosis and from their second diagnosis. HCC, hepatocellular carcinoma; OOPM, only one primary malignancy; SPM, second primary malignancy****.

A total of 40,314 HCC patients diagnosed between 2000 and 2014 were identified from the SEER database. The median follow-up time was 19 months from the initial diagnosis of HCC (interquartile range, 7–47 months). A total of 1,593 patients (3.95%) developed SPMs 2 months after the initial diagnosis of HCC, with a median and maximum follow-up time of 4.58 years and 17.83 years, respectively. The top five most common sites of developing SPMs were the lung and bronchus, prostate, non-hodgkin lymphoma, colon, and breast (**Figure S2**). The demographic and clinicopathological characteristics of the HCC patients with and without SPMs are shown in **Table 1**. We could find that more than half of the patients in the SPM group were aged between 50 and 65 years old, about 70% were male and more than three quarters were white in race. Interestingly, poorer tumor-related characteristics such as larger tumor size, vascular invasion, positive AFP level, poorer tumor grade, and distant extension were associated with a decreased risk of developing SPMs, furthermore, patients who received treatments such as local tumor destruction, hepatectomy, and transplantation were related to a higher risk of developing SPMs. Potentially because patients with poorer tumor-related characteristics without treatment had a chance of death before the development of SPMs. A total of 31,869 patients (79.05%) were dead during follow-up time. The causes of death are shown in **Table 1** and **Figure 1A**, and we could find that 82.84% and 46.37% of the patients died of cancer in the OOPM and SPM groups, respectively.

**Table 1:** Clinicopathological characteristics of patients with HCC with and without SPMs.

Variables	Overall, n (%)	OOPM cohort, n (%)	SPM cohort, n (%)	P value
Total	40,314	38,721 (96.05)	1,593 (3.95)	
Age at initial diagnosis, years				
<50	4,286 (10.63)	4,173 (10.78)	113 (7.09)	<0.001
50–65	22,392 (55.54)	21,493 (55.51)	899 (56.43)	
>65	13,636 (33.82)	13,055 (33.72)	581 (36.47)	
Sex				0.303
Male	31,449 (78.01)	30,223 (78.05)	1,226 (76.96)	
Female	8,865 (21.99)	8,498 (21.95)	367 (23.04)	
Race				0.086
White	26,958 (66.87)	25,852 (66.76)	1,106 (69.43)	
Black	5,259 (13.05)	5,068 (13.09)	191 (11.99)	
Other	8,097 (20.08)	7,081 (21.05)	296 (18.58)	
Tumor grade				<0.001
I-II	12,229 (30.33)	11,578 (29.90)	651 (40.87)	
III-IV	3,067 (7.61)	2,958 (7.64)	109 (6.84)	
Unknown	25,018 (62.06)	24,185 (62.46)	833 (52.29)	
Extension				<0.001
Localized	22,506 (55.83)	21,326 (55.08)	1,180 (74.07)	
Regional	10,635 (26.38)	10,347 (26.72)	288 (18.08)	
Distant	3,891 (9.65)	3,841 (9.92)	50 (3.14)	
Unknown	3,282 (8.14)	3,207 (8.28)	75 (4.71)	
Treatment				<0.001
None	26,667 (66.15)	26,004 (67.16)	663 (41.62)	
Local treatment	5,349 (13.27)	5,064 (13.08)	285 (17.89)	
Hepatectomy	4,620 (11.46)	4,356 (11.25)	264 (16.57)	
Transplantation	3,374 (8.37)	2,999 (7.75)	375 (23.54)	
Unknown	304 (0.75)	298 (0.77)	6 (0.38)	
Tumor size, cm				<0.001
0–2	5,294 (13.13)	4,952 (12.79)	342 (21.47)	
2–5	15,813 (39.22)	15,034 (38.83)	779 (48.90)	
>5	12,334 (30.59)	12,014 (31.03)	320 (20.09)	
Unknown	6,873 (17.05)	6,721 (17.36)	152 (9.54)	
Vascular invasion				<0.001
None	21,275 (52.77)	20,203 (52.18)	1,072 (67.29)	
Yes	6,438 (15.97)	6,245 (16.13)	193 (12.12)	
Unknown	12,601 (31.26)	12,273 (31.70)	328 (20.59)	
AFP				<0.001
Negative	6,996 (17.35)	6,651 (17.18)	345 (21.66)	
Positive	18,977 (47.07)	18,299 (47.26)	678 (42.56)	
Unknown	14,341 (35.57)	13,771 (35.56)	570 (35.78)	
Status				<0.001
Dead	31,869 (79.05)	30,821 (79.60)	1,048 (65.79)	
Alive	8,445 (20.95)	7,900 (20.40)	545 (34.21)	
Latency of SPM, years	–	–	2.33 (0.92–4.92)	–
Cause of death				<0.001
First primary tumor	21,506 (67.48)	21,164 (82.84)	342 (32.63)	
Multiple malignancies	144 (0.45)	0 (0.0)	144 (13.74)	
Noncancer cause	9,416 (29.55)	8,874 (14.62)	542 (51.72)	
Unknown	803 (2.52)	783 (2.54)	20 (1.91)	

HCC, hepatocellular carcinoma; OOPM, only one primary malignancy; SPM, second primary malignancy; AFP, alpha-fetoprotein.

### Survival and Cumulative Incidence of SPM

**Figure 2:**
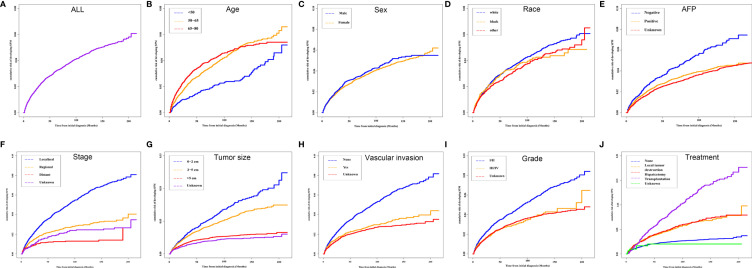
Cumulative incidence curves of SPMs in different subgroups. **(A)** ALL; **(B)** Age; **(C)** Sex; **(D)** Race; **(E)** AFP; **(F)** Stage; **(G)** Tumor size; **(H)** Vascular invasion; **(I)** Grade and **(J)** Treatment. SPM, second primary malignancy; ALL, all patients; AFP, alpha fetoprotein.

The median OS for the OOPM and SPM groups was 18 and 55 months, respectively. The 3-, 5-, and 10-year OS were 32.9%, 23.7%, and 15.7% for the OOPM group, respectively, and for the SPM group, 68.4%, 53.7%, and 31.7%, respectively. As shown in **Figure 1B**, the HCC patients with SPMs had a better OS than those in the OOPM group from the initial diagnosis of HCC; however, these patients had a comparable prognosis with those in the OOPM group after the second diagnosis. The cumulative incidence of SPMs is shown in **Figure 2A**. Regarding death as a competitive event, the 3-, 5-, and 10-year cumulative incidence of developing SPMs were 2.35%, 3.12%, and 4.51%, respectively. Furthermore, the cumulative incidence of developing SPMs in different subgroups classified by various study-, tumor-, and treatment-factors are shown in **Figures 2B–J** and **Table S1**.

### Risk Factors Associated With the Development of SPMs

To avoid the influence of the competitive event of death, the Fine and Gray proportional subdistribution hazards model was used to explore the risk factors of developing SPMs after the initial diagnosis of HCC. As shown in **Table 2**, age at initial diagnosis, extent of disease, treatment, and tumor size were the independent risk factors of developing SPMs. Patients with an older age at the initial diagnosis had a higher risk of developing an SPM, with a subdistribution HR (sdHR) 2.028 for those diagnosed at 65 to 79 years old, compared to those diagnosed younger than 50 years old. Distant and regional extent of disease were associated with a gradual reduction of the risk of developing SPMs than localized disease (sdHR distant 0.468; regional 0.721). Compared to the 0–2 cm group, patients with a larger tumor size had a gradually decreased risk of developing SPMs, with a sdHR of 0.900 and 0.627 for the 2–5 cm and >5 cm subgroups, respectively. In addition, patients who received any treatment were more likely to develop SPMs, with a sdHR of 1.684 (95% CI 1.455–1.948), 2.056 (95% CI 1.747–2.421), and 3.380 (95% CI 2.900–3.939) for those who received local tumor destruction, hepatectomy, and transplantation, respectively.

**Table 2:** Risk factors associated with the development of SPMs for HCC patients after initial diagnosis.

Variables	Multivariate analysis		
Age at initial diagnosis, years	HR	95%CI	P
<50	Reference		
50–65	1.619	1.334–1.966	<0.001
>65	2.028	1.661–2.478	<0.001
Tumor grade			
I-II	Reference		
III-IV	0.829	0.677–1.016	0.07
Unknown	0.975	0.866–1.098	0.68
Extension			
Localized	Reference		
Regional	0.721	0.594–0.875	<0.001
Distant	0.458	0.334–0.628	<0.001
Unknown	0.766	0.554–1.060	0.11
Treatment			
None	Reference		
Local treatment	1.684	1.455–1.948	<0.001
Hepatectomy	2.056	1.747–2.421	<0.001
Transplantation	3.380	2.900–3.939	<0.001
Unknown	0.863	0.384–1.939	0.72
Tumor size, cm			
0–2	Reference		0.11
2–5	0.900	0.790–1.026	<0.001
>5	0.627	0.530–0.743	<0.001
Unknown	0.629	0.503–0.786	
Vascular invasion			
None	Reference		
Yes	0.845	0.714–1.001	0.051
Unknown	0.941	0.768–1.021	0.56
AFP			
Negative	Reference		
Positive	0.895	0.785–1.021	0.098
Unknown	0.957	0.834–1.097	0.53

HCC, hepatocellular carcinoma; AFP, alpha fetoprotein; SPM, secondary primary malignancy.

### Development and Validation of a Competing-Risk Nomogram

**Figure 3:**
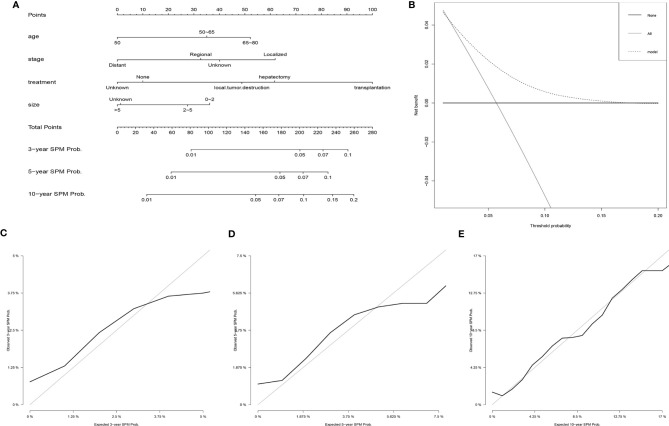
Establishment and validation of a competing-risk nomogram to predict the 3-, 5-, and 10-year probability of developing SPMs for HCC patients after initial diagnosis. **(A)** The competing-risk nomogram for predicting the 3-, 5-, and 10-year probability of developing SPMs for HCC patients after initial diagnosis. **(B)** DCA for assessing the clinical net benefit of the nomogram. Calibration curves of the competing-risk nomogram for predicting the 3- **(C)**, 5- **(D)**, and 10-year **(E)** probability of developing SPMs for HCC patients after initial diagnosis. HCC, hepatocellular carcinoma; SPM, second primary malignancy; DCA, decision curve analysis.

Based on the results of the Fine and Gray proportional subdistribution hazards model analysis, a competing-risk nomogram was established to predict the 3-, 5-, and 10-year probability of developing SPMs for HCC patients after the initial diagnosis (**Figure 3A**). The C-index of the nomogram was 0.677 (95% CI 0.676–0.678), which showed that it had a moderate performance. The calibration curves also suggested that the nomogram performed well, in which the calibration curves were in good concordance with the 45° diagonal line (**Figures 3C–E**). In addition, DCA was used to evaluate the clinical usefulness and net benefit of the competing-risk nomogram, and the results suggested that compared to those in the two alternative scenarios: no-screening and all-screening scenarios, the clinical net benefit of the competing-risk nomogram was higher than that in the other two scenarios in a wide range of threshold probabilities (ranged from 1% to 17%, **Figure 3B**).

**Figure 4:**
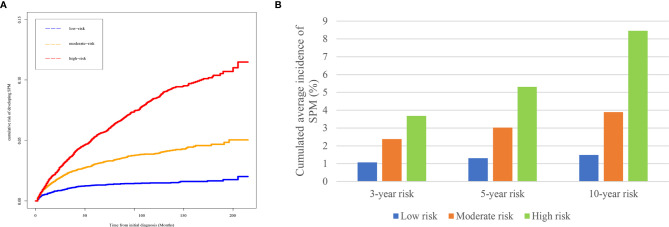
Risk stratification ability of the competing-risk nomogram. **(A)** Cumulative incidence curves of SPMs among low-, moderate-, and high-risk survivors. **(B)** Cumulative 3-, 5-, and 10-year incidence of developing SPMs among low-, moderate-, and high-risk survivors. HCC, hepatocellular carcinoma; SPM, second primary malignancy.

Based on the risk scores gained from the competing-risk nomogram for each HCC patients, the included patients in the SEER dataset could be divided into three risk groups: low-, moderate-, and high-risk groups. As shown in **Figure 4**, compared to those in the low-risk group, patients in the moderate- and high-risk groups exhibited a significantly higher cumulative incidence of SPMs after the initial diagnosis of HCC.

## Discussion

The number of cancer survivors is growing; in the U.S., it was reported that the number of cancer survivors was estimated to reach 26.1 million by 2040, which approximately accounted for 6.9% of the total population (22). Due to the special disease history, many factors are threats for the health of the survivors, such as the recurrence of cancer (23) and treatment-related toxicities (24); meanwhile, SPMs, with a growing incidence, is becoming a threat on the health of cancer survivors (25, 26). It was reported that the incidence of SPMs ranged from 1% to 16% according to various cancer types (27). However, cancer survivors were frequently excluded from cancer clinical trials, and little is known about their characteristics, risk factors, and survival (28–30). Consequently, exploring the characteristics of SPMs, identifying the risk factors associated with the development of SPMs and screening patients at high risk of developing SPMs are of both a clinical and public health importance. In this study, we identified 1,593 patients (total population: 40,314) who developed SPMs after the initial diagnosis of HCC. In the further analysis, we found that compared to those in the OOPM group, patients with SPMs had better tumor-related characteristics and had a higher proportion of receiving tumor-related treatment. Furthermore, using the Fine-Gray proportional subdistribution hazards model, risk factors associated with the development of SPMs were explored and a competing-risk nomogram was established to predict the 3-, 5-, and 10-year probability of developing SPMs for HCC patients after the initial diagnosis. The C-index, calibration curves, and DCA all showed that the nomogram had a good performance. To our knowledge, this study provided the largest population-based cohort study of SPM incidence in HCC patients.

The incidence of SPMs in HCC patients was 3.95% in this study. In contrast, the incidence of SPMs in a previous US population-based study which included patients from 1992 to 2011 in the SEER database was reported to be 2.83% (13). Meanwhile, the incidence of SPMs was various in previous studies of different countries, which was around 3.5%–7.5% in the USA (10, 31), 2.4% in Spain (32), 1.63%–8.0% in China (12, 33), and 0.7%–1.9% in Japan (34, 35). This might be influenced by the difference in the demographic characteristics, etiologies, tumor characteristics, and treatment strategies, which were also analyzed in our study. In the analysis of the risk factors of developing SPMs among different subgroups, we could find that the development of SPMs was greatly influenced by the factors including vascular invasion, stage, treatment, tumor size, sex, and age at initial diagnosis. It is clear that the long-term results of HCC survivors could be different depending on these factors.

The top five most common sites of developing SPMs were the lung and bronchus, prostate, non-Hodgkin lymphoma, colon, and breast. Consequently, for HCC survivors at a high risk of developing SPMs, regular and long-term surveillance for lung and bronchus, prostate (male patients), and breast (female patients) was necessary, and there might be a need for a discussion by the multidisciplinary team. Furthermore, in the survival analysis, we found that the patients in the SPM group had a better survival than those in the OOPM group. However, for HCC patients with SPMs after the second diagnosis, they had a comparable survival with those in the OOPM group. We also found that 82.84% patients died of cancer in the OOPM group while only 46.37% patients in the SPM group died of cancer. We thought that the main reason was that patients in the OOPM group were associated with poorer tumor-related characteristics and had a low proportion of receiving tumor-related treatment, consequently, these patients were more likely to have a poorer prognosis and die of cancer.

Since a large proportion of patients died before the development of SPMs, it is necessary to conduct a proper statistical method to handle this competitive event. Using the Fine and Gray proportional subdistribution hazards model, we explored the risk factors of developing SPMs from the demographic and clinical variables. The multivariate analysis suggested that patients with SPMs tend to have an older age at the initial diagnosis, a local or regional disease, and a smaller tumor size. These variables have been found to be associated with an increased risk of developing SPMs in other cancers (36–38). Furthermore, patients who received any tumor-related treatment had a higher risk of developing SPMs, especially for those receiving transplantation. Several reasons could explain our results: first, the immune system has been reported to be related to cancer development, and it was reported that older age was associated with immunity decreasing (“immunosenescence”), which might contribute to an increased cancer incidence (39). Second, since HCC was a malignancy with a poor prognosis, HCC survivors with better prognostic factors were believed to have a better survival, and thus more likely to have an adequate time to develop SPMs (4, 22). Third, for patients who received treatment for HCC, they might have a higher susceptibility to tumorigenesis, especially for those who received liver transplantation, the administration of immunosuppressive therapy might contribute to the development of SPMs (40–42).

Considering the elevated risk of developing SPMs after the initial diagnosis of HCC, a competing-risk nomogram was established for the purpose of improving guideline surveillance and further management of HCC survivors. To our knowledge, this is the first model for the prediction of developing SPMs for HCC after the initial diagnosis, and our nomogram could be useful to predict the 3-, 5-, and 10-year probability of developing SPMs for HCC survivors. Furthermore, the performance of our nomogram was also demonstrated well using the C-index and calibration curves, and DCA further suggested that the nomogram had a superior net benefit in clinical scenarios. Consequently, our nomogram is potentially useful for doctors to screen HCC survivors who are at a high risk of developing SPMs and could contribute to the further management of SPMs.

There are several limitations in our study. First, due to the nature of the SEER database, several important cancer-related risk factors, such as lifestyle, alcohol and tobacco consumption, and family history were unavailable; meanwhile, for HCC, several well-known prognostic factors, including cirrhosis, portal hypertension, postoperative complication, and microenvironment-related immune and inflammatory markers (43, 44) were also unavailable. These might lead to a bias of the results. However, our study still found several risk factors of developing SPMs for HCC and our predictive model showed the ability to discriminate patients who are at a high risk of developing SPMs. Second, the cumulative incidence of SPMs could be overestimated when extrahepatic metastasis was regarded as SPMs or the initial simultaneous cancers could not be distinguished using a 2-month latency exclusion. Certainly, the diagnosis time of an SPM might not be the accurate time when it occurred, and these could be solved with the advancement of detection technology and regular surveillance. Third, although our nomogram had a good performance, further validation with external populations is still needed.

## Conclusions

In conclusion, we demonstrated that age at diagnosis, tumor stage, tumor size, and treatment were associated with an increased risk of developing SPMs for HCC patients after the initial diagnosis. Afterwards, a reliable competing-risk nomogram was established to predict the 3-, 5-, and 10-year probability of developing SPMs for HCC survivors. Our findings could be useful to clinicians for the surveillance and management of HCC patients. Further exploration involving more patients and more risk factors should be performed, and better surveillance should be conducted for HCC patients who are at a high risk of developing SPMs.

## Data Availability Statement

The raw data supporting the conclusions of this article will be made available by the authors, without undue reservation.

## Author Contributions

GY, WS, and JL contributed to the conception and design of the study. JK, WS, JC, and GL collected the details of the patients and extracted the data. JK, YL, and JC analyzed the data. JK, GY, and GL drafted the manuscript. JL, WS, JC, and YL contributed with a critical revision of the manuscript. All authors contributed to the article and approved the submitted version.

## Funding

This study was supported by the National Natural Science Foundation of China (No. 81373172, 81770646).

## Conflict of Interest

The authors declare that the research was conducted in the absence of any commercial or financial relationships that could be construed as a potential conflict of interest.

## Publisher’s Note

All claims expressed in this article are solely those of the authors and do not necessarily represent those of their affiliated organizations, or those of the publisher, the editors and the reviewers. Any product that may be evaluated in this article, or claim that may be made by its manufacturer, is not guaranteed or endorsed by the publisher.

## Supplementary Material

Supplementary Figure 1 Work flow of patient selection from the Surveillance, Epidemiology, and End Results (SEER) research database.

Supplementary Figure 2 Top 10 most common sites of developing a secondary primary malignancy in patients with hepatocellular carcinoma.
