# Supplementary figures and images for: Second Primary Malignancies in Patients With Hepatocellular Carcinoma: A Population-Based Analysis
Source: Front Oncol. 2021 Aug 23;11:713637. doi: 10.3389/fonc.2021.713637 (PMC8420091; doi:10.3389/fonc.2021.713637)

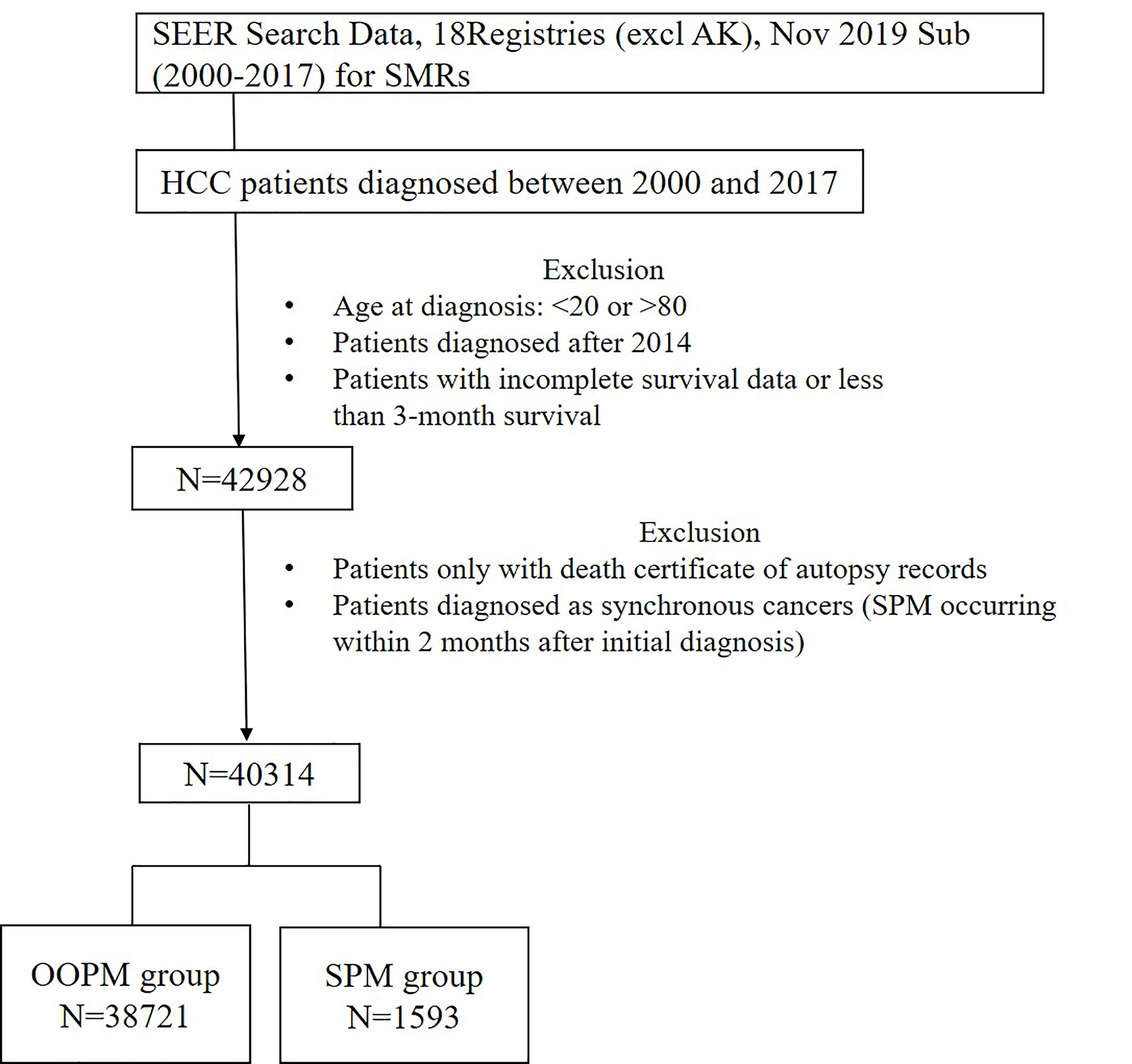

Supplement: Supplementary Figure 1 — Work flow of patient selection from the Surveillance, Epidemiology, and End Results (SEER) research database. [file Image_1.tif]

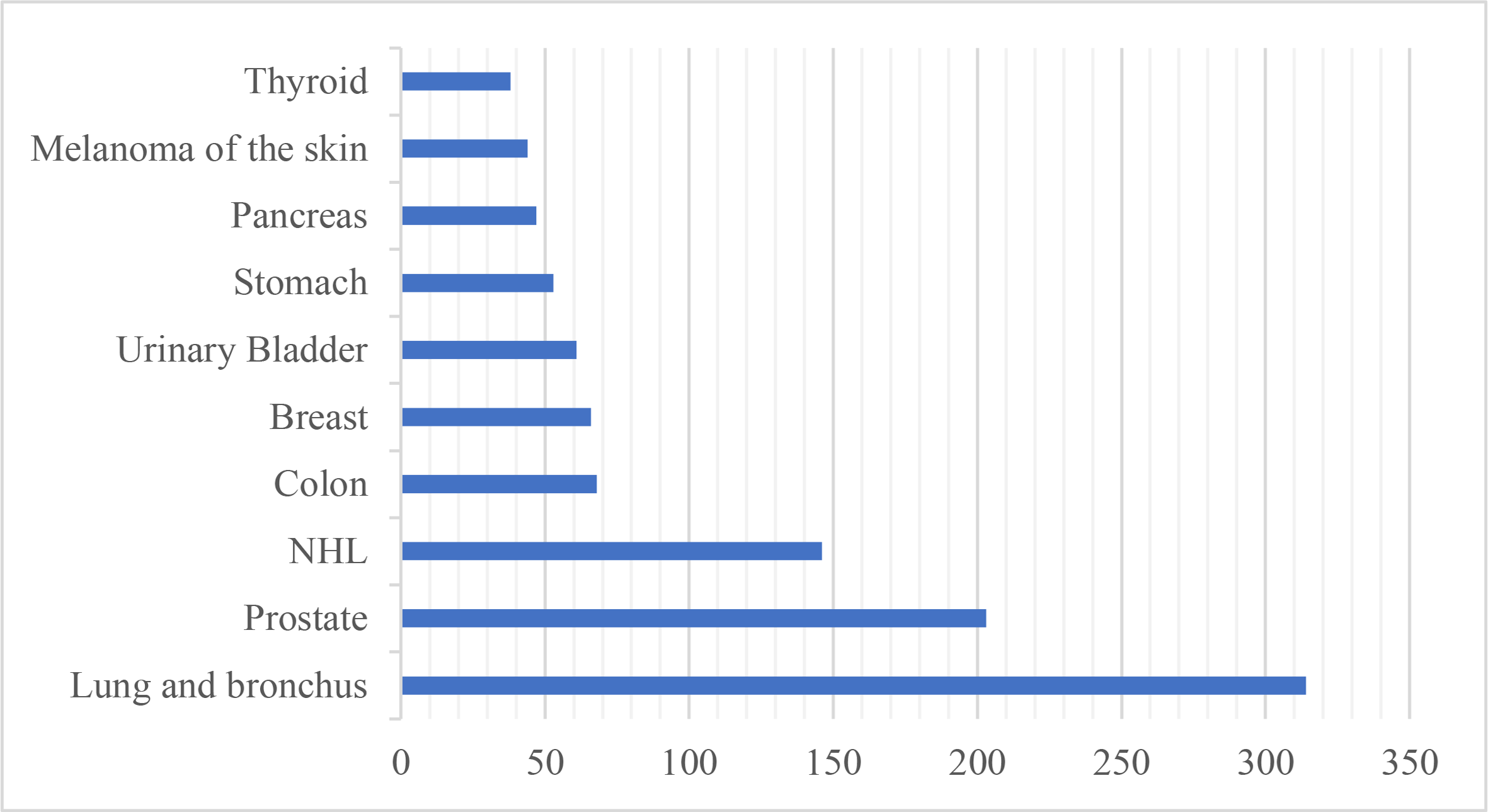

Supplement: Supplementary Figure 2 — Top 10 most common sites of developing a secondary primary malignancy in patients with hepatocellular carcinoma. [file Image_2.tif]
