# Supplementary material for: Second Primary Malignancies in Patients With Hepatocellular Carcinoma: A Population-Based Analysis
Source: Front Oncol. 2021 Aug 23;11:713637. doi: 10.3389/fonc.2021.713637 (PMC8420091; doi:10.3389/fonc.2021.713637)
Supplement: Supplementary file 3 [file Table_1.docx]

Table S1 Cumulative incidence of SPMs in 1, 3, 5 and 10 year among different subgroups for HCC survivors

|  | 1 year | 3 year | 5 year | 10 year |
| --- | --- | --- | --- | --- |
| Overall | 1.13% | 2.35% | 3.12% | 4.51% |
| Age at initial diagnosis, years | |  |  |  |
| < 50 | 0.61% | 1.13% | 1.71% | 2.44% |
| 50-65 | 0.92% | 2.28% | 3.08% | 4.74% |
| > 65 | 1.45% | 2.84% | 3.74% | 4.91% |
| Sex |  |  |  |  |
| Male | 1.15% | 2.48% | 3.30% | 4.77% |
| Female | 1.12% | 2.31% | 3.11% | 4.45% |
| Race |  |  |  |  |
| White | 1.03% | 2.44% | 3.26% | 4.68% |
| Black | 1.24% | 2.28% | 3.23% | 4.26% |
| Other | 1.09% | 2.09% | 2.75% | 4.17% |
| Tumor grade |  |  |  |  |
| I-II | 1.32% | 2.90% | 3.86% | 5.93% |
| III-IV | 1.11% | 2.14% | 2.74% | 3.97% |
| Unknown | 0.99% | 2.08% | 2.83% | 3.87% |
| Extension |  |  |  |  |
| Localized | 1.35% | 2.91% | 4.07% | 6.11% |
| Regional | 0.99% | 1.88% | 2.32% | 2.97% |
| Distant | 0.67% | 1.15% | 1.21% | 1.30% |
| Unknown | 0.61% | 1.45% | 1.86% | 2.46% |
| Treatment |  |  |  |  |
| None | 0.94% | 1.82% | 2.29% | 2.77% |
| Local treatment | 1.59% | 3.10% | 4.16% | 6.43% |
| Hepatectomy | 1.61% | 3.16% | 4.34% | 6.47% |
| Transplantation | 1.19% | 4.29% | 6.84% | 12.2% |
| Unknown | 0.98% | 1.66% | 1.66% | 2.02% |
| Tumor size, cm |  |  |  |  |
| 0-2 | 1.40% | 3.31% | 4.69% | 7.87% |
| 2-5 | 1.20% | 2.80% | 3.90% | 5.74% |
| > 5 | 0.95% | 1.83% | 2.26% | 2.78% |
| Unknown | 0.73% | 1.44% | 1.87% | 2.33% |
| Vascular invasion |  |  |  |  |
| None | 1.22% | 2.84% | 3.96% | 5.96% |
| Yes | 0.95% | 1.89% | 2.41% | 3.23% |
| Unknown | 0.77% | 1.76% | 2.18% | 2.81% |
| AFP |  |  |  |  |
| Negative | 1.40% | 2.98% | 4.15% | 6.32% |
| Positive | 1.16% | 2.34% | 3.11% | 4.24% |
| Unknown | 0.95% | 2.05% | 2.74% | 4.02% |

Note: HCC, hepatocellular carcinoma; SPM, secondary primary malignancy.
